# Iterative analytic extension in tomographic imaging

**DOI:** 10.1186/s42492-021-00099-5

**Published:** 2022-02-01

**Authors:** Gengsheng L. Zeng

**Affiliations:** 1 Department of Computer Science, Utah Valley University, Orem, UT 84058 USA; 2 Department of Radiology and Imaging Sciences, University of Utah, Salt Lake City, UT 84108 USA

**Keywords:** Analytic continuation, Entire function, Iterative projections onto convex sets algorithm, Image reconstruction, Limited angle tomography

## Abstract

If a spatial-domain function has a finite support, its Fourier transform is an entire function. The Taylor series expansion of an entire function converges at every finite point in the complex plane. The analytic continuation theory suggests that a finite-sized object can be uniquely determined by its frequency components in a very small neighborhood. Trying to obtain such an exact Taylor expansion is difficult. This paper proposes an iterative algorithm to extend the measured frequency components to unmeasured regions. Computer simulations show that the proposed algorithm converges very slowly, indicating that the problem is too ill-posed to be practically solvable using available methods.

## Introduction

In medical or industrial imaging, a stable image reconstruction depends on sufficient data acquisition. The data acquisition geometry required for a stable reconstruction is different from that demanded theoretically. As pointed out in Naterer’s book [1], a stable reconstruction in parallel-beam imaging requires the angular detector coverage of 180°. However, it is theoretically possible to perform limited-angle tomography, for example, with a smaller angular coverage of just 10°. As stated in ref. [1], it is an extremely ill-posed problem to reconstruct an image from a very small angular coverage. In other words, it is practically impossible to stably reconstruct an image with a very small angular coverage with noisy measurements. The ill-condition characters were mathematically established by studying the spectrum of the singular values of the limited data tomography [1].

The theoretical foundation of image reconstruction with a very small angular coverage is the Paley-Wiener theorem (see Theorem 7.2.1 in ref. [2]) and analytic extension (or continuation) (see Chapter V in ref. [3]). The Paley-Wiener theorem states that if a function has a finite support, then its Fourier transform is an entire function. In complex analysis, an entire function, also known as an integral function, is a complex-valued function that is holomorphic (analytic) on the whole complex plane. An entire function *F* has a power series expansion (i.e., Taylor expansion) in *z* that converges at every finite point in the complex plane:

1 $$ F(z)=\sum \limits_{k=0}^{\infty}\frac{F^{(k)}\left({z}_0\right)}{k!}{\left(z-{z}_0\right)}^k $$

where *z*_0_ is any complex number. This Taylor expansion plays an important role in analytic extension. The concept of analytic extension implies that if an entire function *F*(*z*) is known at a point *z*_0_ and its neighborhood, its Taylor series is known and consequently *F*(*z*) is determined on entire complex plane by this Taylor series expansion. This theory looks good on paper but is pretty much useless in practice because we do not know how to obtain the various orders of the derivatives of *F*(*z*) exactly, for the finite difference approximation is not accurate enough.

Let *f*(*x*) be a one dimensional (1D) function with a finite support on [−*A*, *A*]. Its 1D Fourier transform is

2 $$ F\left(\omega \right)=\underset{-A}{\overset{A}{\int }}f(x){e}^{- i\omega x} dx $$

where *ω* is a real number representing the radian frequency, and *F*(*ω*) is a complex function with a single real variable *ω*. If we replace *ω* by a complex variable *z = u + iv*, Formula (2) becomes an entire function defined on the complex plane:

3 $$ F(z)=\underset{-A}{\overset{A}{\int }}f(x){e}^{- izx} dx $$

It can be verified (see Appendix) that the Cauchy–Riemann equations are satisfied and *F*(*z*) in Formula (3) is indeed an entire function on the complex plane [4]. If *F*(*z*) is measured at a point’s neighborhood, for example, at *z* = 0, *F*(*z*) is determined on the whole complex plane, and hence *f*(*x*) is determined (in theory).

**Fig. 1:**
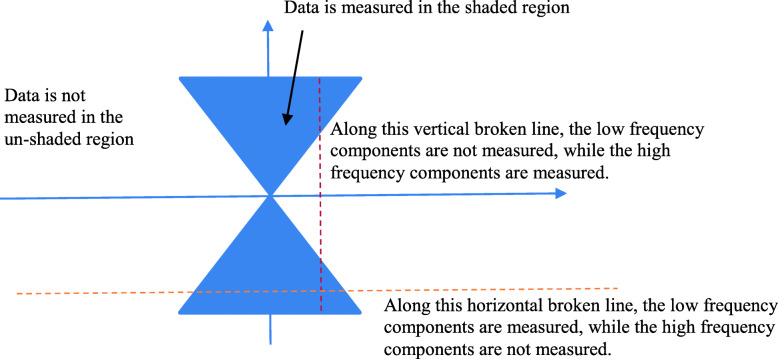
A situation of limited angle data acquisition is shown in the 2D Fourier domain. The shaded area represents measured region. If a horizontal line is considered, the low frequencies are measured, while the high frequencies are not. On the other hand, if a vertical line is considered, the high frequencies are measured, while the low frequencies are not

Figure 1 shows a two dimensional (2D) Fourier space. The shaded wedge areas are measured. The center of the Fourier space is the low frequency region. The outer area is the high frequency region. Along the horizontal dashed line shown in Fig. 1, the lower frequency components are measured, while the higher frequency components are not measured. On the other hand, if we draw a vertical line (not passing through the origin), the higher frequency components are measured, while the lower frequency components are not measured along this vertical line.

In tomographic imaging, the line integrals along one direction of a 2D section can be considered as a 1D function with a finite support. A collection of such 1D functions at various angles forms the sinogram. We take the 1D Fourier transform row-by-row of the sinogram and map them to the 2D Fourier space according to the Fourier Slice Theorem (i.e., the Central Slice Theorem) [5]. The 2D Fourier space is completely covered if the scanning angular coverage is 180°. If the angular coverage is a smaller, only wedge-shaped regions are measured in the 2D Fourier space as shown in Fig. 1, where the dashed line illustrates the 1D Fourier transform *F* of a sinewave-encoded 1D line-integrals with a finite support. The 1D function *F* is an entire function and is partially available. We wish to estimate the unmeasured parts of *F*. In a row-by-row manner, the whole 2D Fourier space is estimated, and the spatial domain counterpart can be reconstructed. In this case, the low-frequency components are measured, and the high-frequency components are to be estimated.

Alternatively, we can draw a vertical. We will have a different estimation problem. The high-frequency components are measured, and the low-frequency components are to be estimated.

## Methods

### Problem statement

The problem we are going to solve in this paper is described as follows. We assume that the spatial-domain object has a finite support. The Fourier transform of this object is determined by is partial measurements in the Fourier domain. The goal is to estimate the frequency components in the unmeasured regions.

### Proposed algorithm

Since the Taylor expansion method is not practical, we need to develop something more practical. We are inspired by an iterative algorithm in the theory of antenna synthesis [6] and propose an iterative algorithm to extend *F* from measured region to unmeasured regions. The algorithm is an ad hoc “projections onto convex sets” (POCS) algorithm [7]. The POCS algorithms are a family of iterative algorithms that bounce back and forth between different domains, to enforce the solution to satisfy the restrictions in every domain.

The proposed algorithm is an iterative procedure, alternating between the Fourier domain and the spatial domain in each iteration. In the spatial domain, this object is denoted as *f*. The Fourier transform of this object is denoted as *F* in the Fourier domain.

The partial measurements of the object are acquired in the Fourier domain. In the proposed iterative algorithm, an initial condition is set in the Fourier domain by assuming the unmeasured data be zero. At this point, the object is referred to as “projecting onto the Fourier domain,” which implies that the Fourier-domain constraints are satisfied. This is the halfway through the first iteration.

The second half of the first iteration is performed in the spatial domain. We take the inverse Fourier transform of the Fourier-domain data prepared in the first half, to obtain an image in the spatial domain. After the inverse Fourier transform, the spatial-domain image is complex, having both the real part and the imaginary part. We require that the spatial-domain image is non-negative and has a finite support. To make the image non-negative, we take the Euclidean norm of the data, which is the square-root of the sum of the real part square and the imaginary part square. If the support is known, set the image values to zero outside the support. At this point, the object is referred to as “projecting onto the spatial domain,” which implies that the spatial-domain constraints are satisfied. This is the second halfway through the first iteration. The first iteration is completed.

We must realize that the second half way’s action may alter the results from the action in the first half. In other words, the Fourier-domain requirements may be no longer satisfied. This is the reason that the second iteration is required.

After the first half of the second iteration, the Fourier-domain constraints are satisfied. Unfortunately, the spatial-domain requirements may be somewhat destroyed. Then the second half is performed for the second iteration, to make the spatial-domain constraints satisfied.

Hopefully, after many iterations, both the Fourier-domain constraints and the spatial-domain constraints are somewhat satisfied.

In summary, the suggested algorithm consists of the following steps with spatial-domain function *f*(*x*) and Fourier-domain counterpart *F*(*ω*). Initially, the Fourier-domain function *F*(*ω*) is set to 0.

Step 1: In the Fourier domain, enforce *F*(*ω*) to the known measured value if *F*(*ω*) is measured at *ω*. Otherwise, the value of *F*(*ω*) remains the same as before.

Step 2: Take the inverse Fourier transform of the function *F*(*ω*) obtained in step 1. Set *f*(*x*) to the norm of the inverse Fourier transform of *F*(*ω*).

Step 3: Take the Fourier transform of the *f*(*x*) obtained in step 2, obtaining a new Fourier-domain function *F*(*ω*). Go back to step 1.

[Repeat the above 3 steps].

**Fig. 2:**
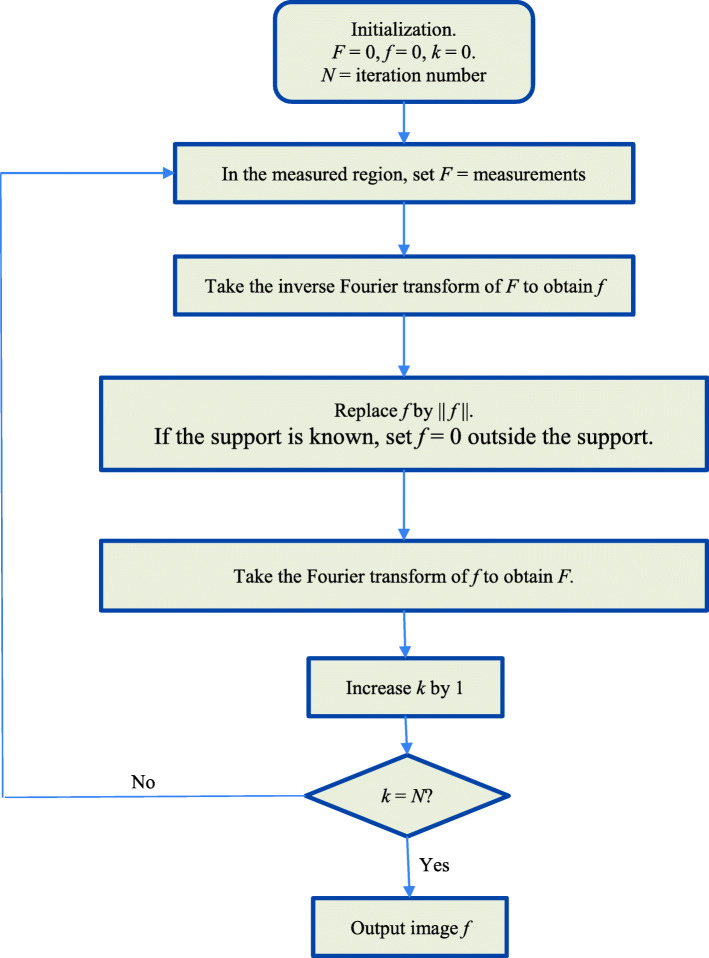
The flow chart of the proposed iterative algorithm

Step 1 enforces the Fourier-domain measurements. Step 2 enforces the spatial-domain real and non-negativity constraints. This algorithm is much easier to implement than the Taylor expansion method. The Fourier transform and the inverse Fourier transform can be readily implemented by the fast Fourier transform and the inverse fast Fourier transform [8]. Figure 2 shows a flowchart for the proposed algorithm.

## Results

**Fig. 3:**
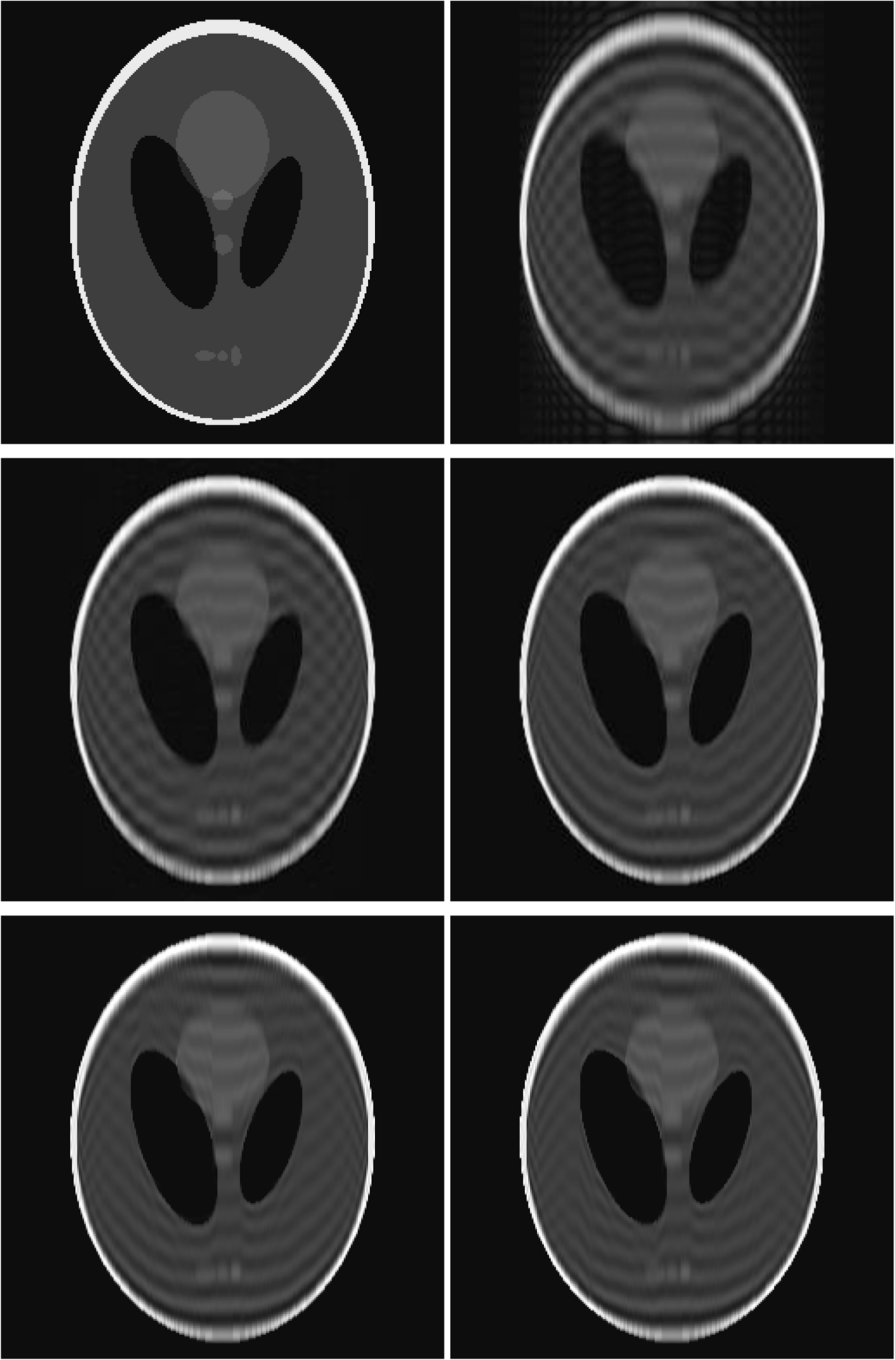
Reconstruction of the Shepp-Logan phantom with noiseless data. Row 1: (L) the true phantom; (R) the result with the initial condition. Row 2: (L) the result with 10^2^ iterations; (R) the result with 10^4^ iterations. Row 3: (L) the result with 10^6^ iterations; (R) the result with 10^8^ iterations

A noiseless 2D Shepp-Logan phantom computer simulation was performed [9]. The image size was 256 × 256. The simulation was only performed in the vertical (*y*) direction. The measured frequency components with absolute index values from zero up to 15. Various iteration numbers were tested. The images for the noiseless study are shown in Fig. 3. The image with the initial condition was obtained by using the 15 lowest frequency components. The restored images in the second row were obtained by the proposed iterative algorithm. The corresponding mean squared errors (MSEs) for the images are listed in Table 1. No noise was added to the measurements in Fig. 3.

**Table 1:** MSEs for the Shepp-Logan phantom study without noise. Computation times are also reported

Iteration number	MSE	Time (s)
Initial	594.98	
10	433.4856	27.978506
10^2^	341.7619	28.284021
10^3^	286.0546	27.900569
10^4^	253.1430	36.472132
10^5^	232.3008	53.552202
10^6^	218.0490	283.494466
10^7^	207.5428	2551. 410,758
10^8^	199.8592	24,890.904604

**Fig. 4:**
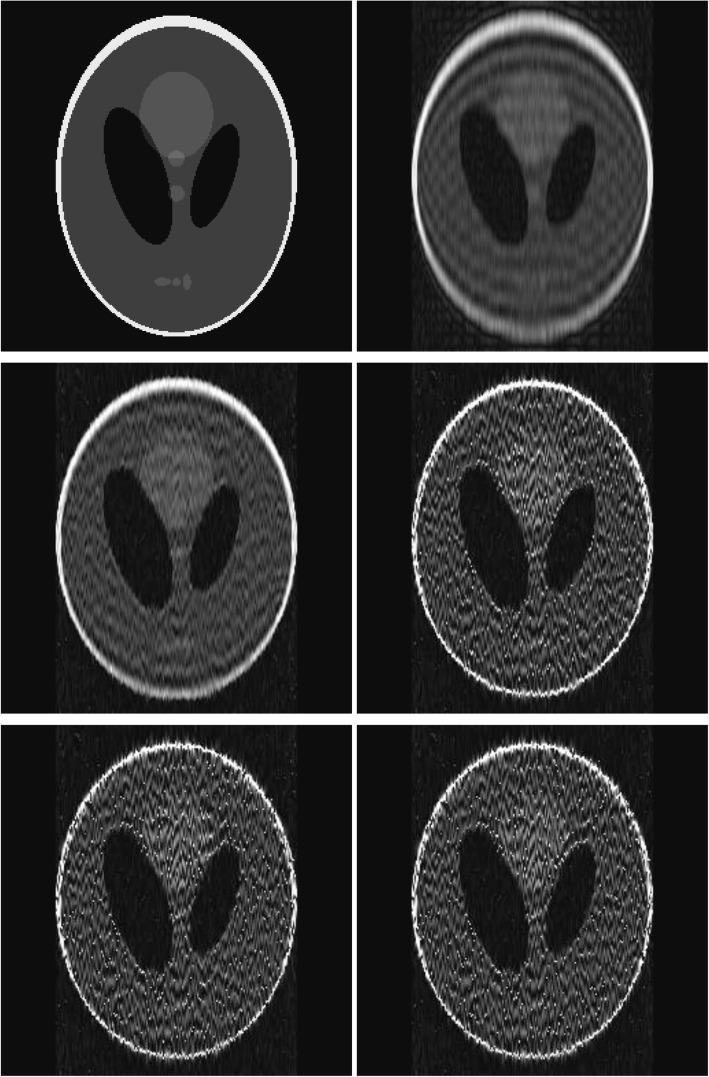
Reconstruction of the Shepp-Logan phantom with noisy data. Row 1: (L) the true phantom; (R) the result with the initial condition. Row 2: (L) the result with 10^2^ iterations; (R) the result with 10^4^ iterations. Row 3: (L) the result with 10^6^ iterations; (R) the result with 10^8^ iterations

Noisy study results with Gaussian white noise are shown in Fig. 4. The signal-to-noise ratio was set at ten. These noisy images indicate that the reconstruction problem with incomplete data is extremely unstable and may not find any applications in the real world.

The algorithm was very slow to converge. In other words, we do not see many changes or improvements between iterations. Due to incomplete data, the images did not converge to the true image. The very slow convergence trend can also be observed from the MSE values in Tables 1 and 2, for the noiseless and noisy cases, respectively. When the iteration was as high as 10^8^, the algorithm was still not converged. This slow convergence is an indicator that the inverse problem is severely ill-posed.

**Table 2:** MSEs for the Shepp-Logan phantom study with noise. Computation times are also reported

Iteration number	MSE	Time (s)
Initial	625.90	
10	465.5	27.634184
10^2^	460.4	27.595421
10^3^	1044.5	27.803038
10^4^	2269.4	31.491420
10^5^	3520.0	57.319462
10^6^	4251.0	319.610876
10^7^	4049.3	3040.756353
10^8^	4081.7	29,169.871348

**Fig. 5:**
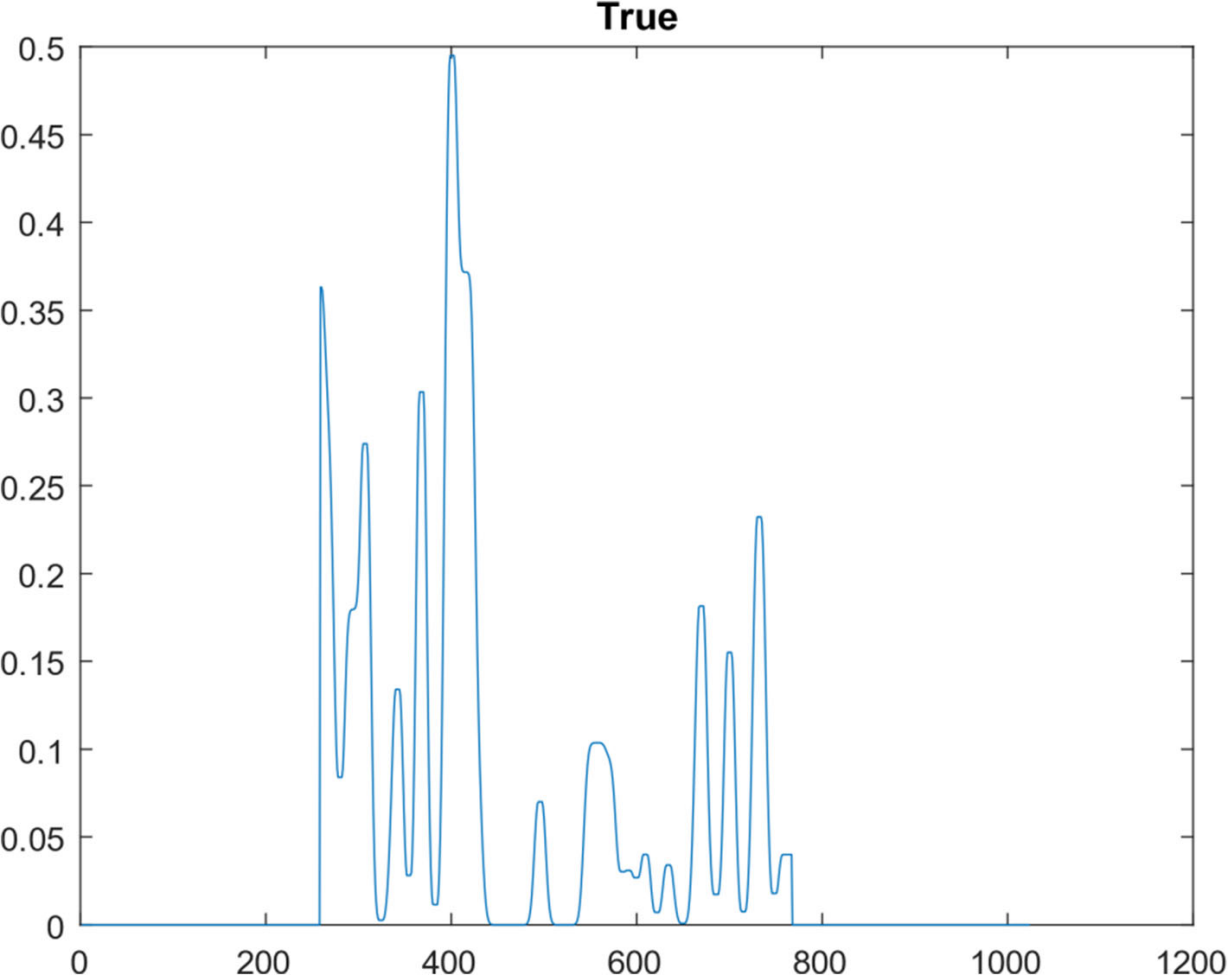
The true ‘random’ function

**Fig. 6:**
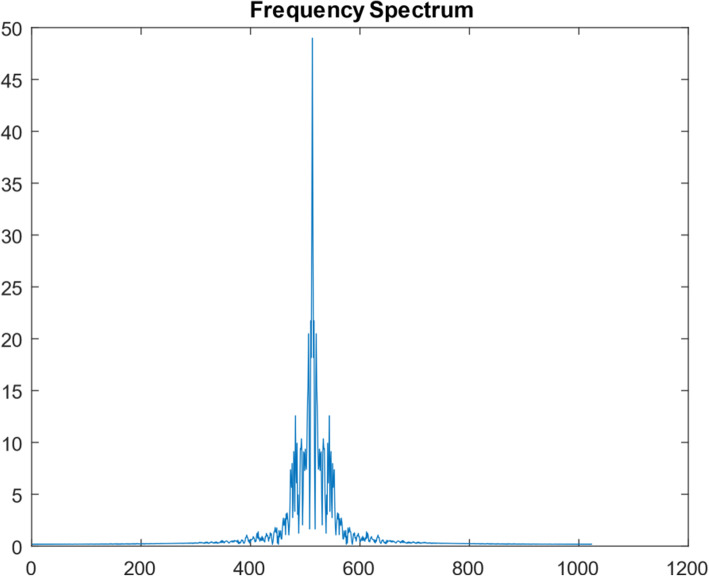
The frequency spectrum of the true ‘random’ function

**Fig. 7:**
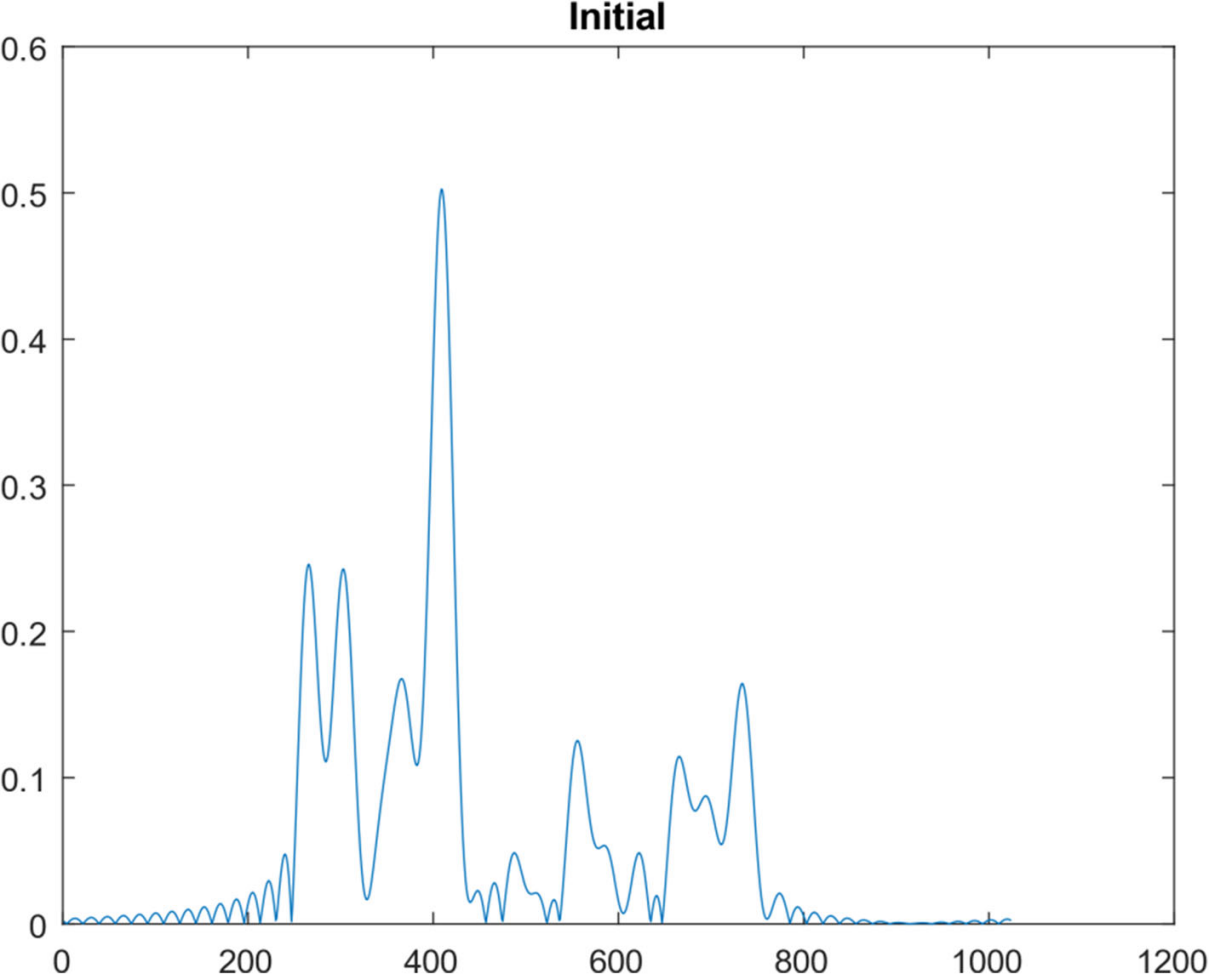
The spatial-domain function obtained from the lowest 30 frequency components of the ‘random’ function

**Fig. 8:**
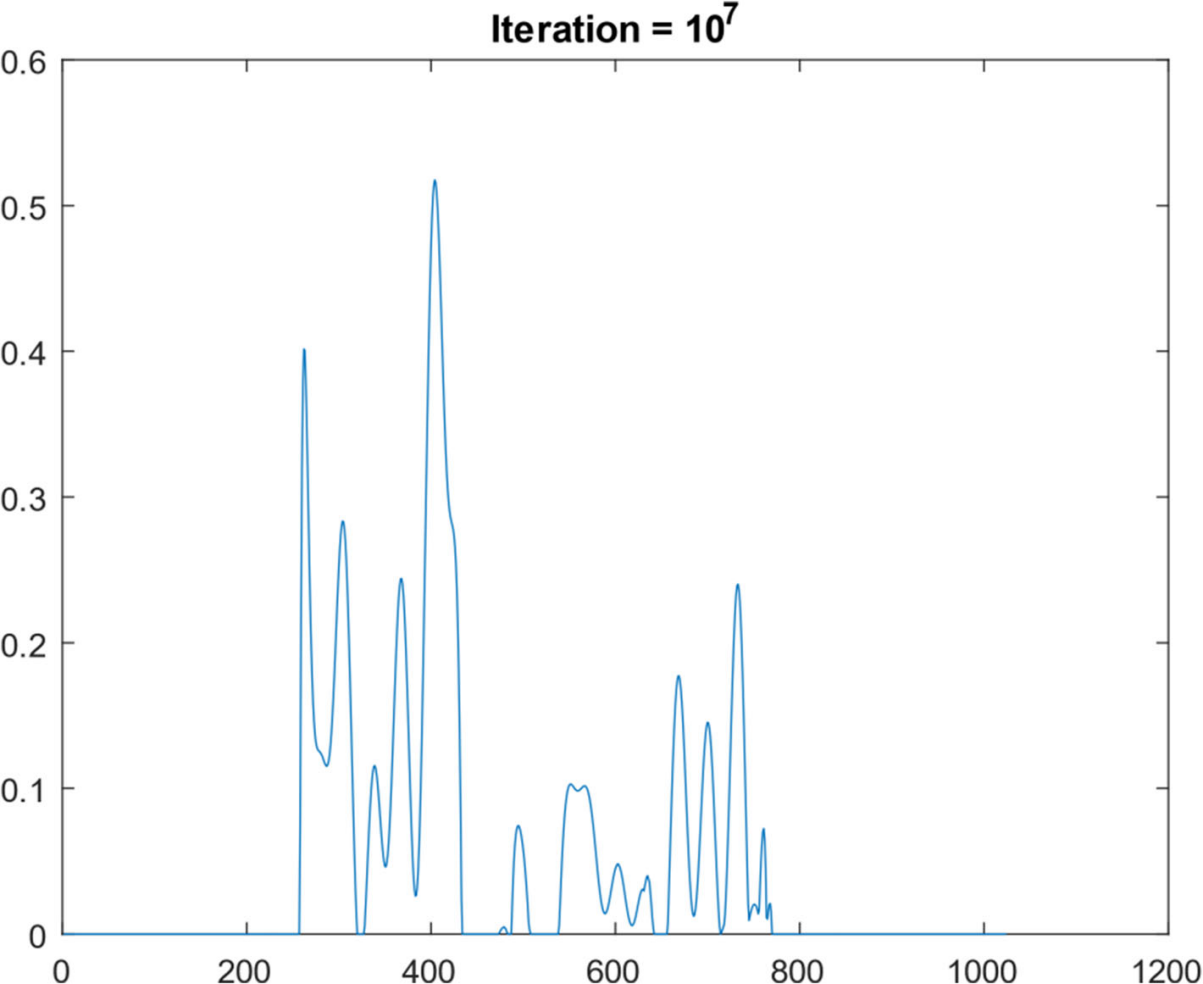
Restored the ‘random’ function after 10^7^ iteration of suggested algorithm

A 1D function simulation was performed with 10^8^ iterations. This 1D function was ‘randomly’ generated, consisting of 1024 points, as shown in Fig. 5. Its frequency spectrum (i.e., the magnitude of its Fourier transform) is shown in Fig. 6. In the frequency domain, 30 lowest frequency points were measured. We first took the 1024-point Fourier transform of the simulated true 1D ‘random’ function. The Fourier transform of the initial condition (shown as Fig. 7) was obtained by setting all 1024 – 30 = 994 frequency components to zero except the 30 lowest frequency points. The result after 10^7^ iterations is shown in Fig. 8. The curves in Figs. 5, 7, and 8 are in the spatial domain; the curve in Fig. 6 is in the frequency domain.

**Fig. 9:**
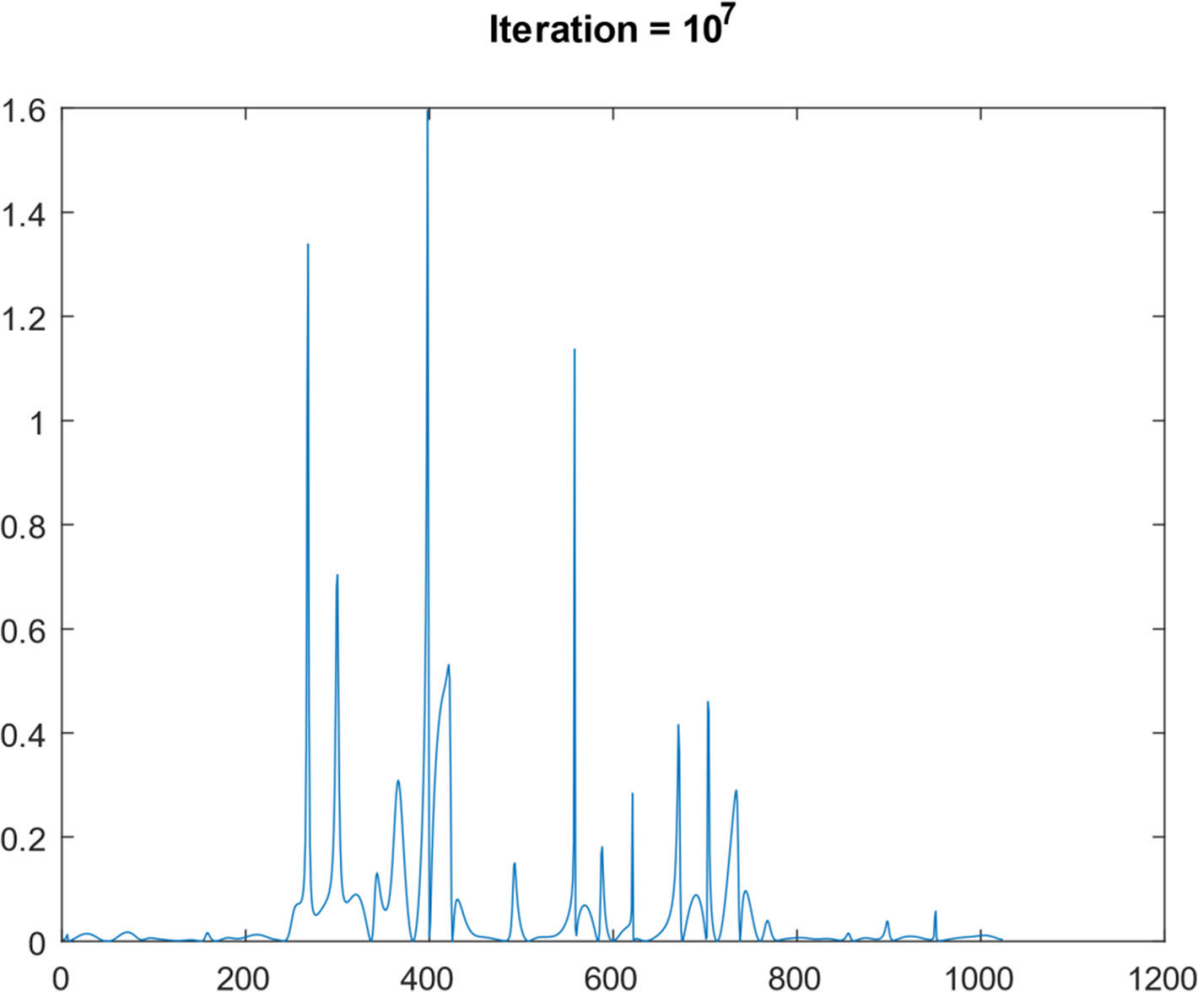
Restored the ‘random’ function after 10^7^ iteration of suggested algorithm using noisy data

No noise was added to the 1D data. The convergence rate was very slow, as indicated by the MSEs are listed in Table 3. However, one can observe some improvements from the initial condition to the 10^8^th iteration result. If a little noise is added (with a signal-to-noise ratio being ten), the results (as shown in Fig. 9) from high iterations are very noisy and not usable.

**Table 3:** MSEs for the 1D ‘random’ curve study with noiseless data

Iteration number	MSE
Initial	1.5 × 10^−3^
10^2^	9.3 × 10^−4^
10^4^	6.8 × 10^−4^
10^6^	3.7 × 10^− 4^
10^8^	3.5 × 10^−4^

Another 1D function simulation was also performed with 10^7^ iterations. This different 1D function was a Gaussian curve, consisting of 1024 points. Figures 10, 11, 12, 13 and 14 are the counterparts of Figs. 5, 6, 7, 8 and 9, respectively. Table 4 is the counterpart of Table 3. Table 4 reports much better results than Table 3.

**Fig. 10:**
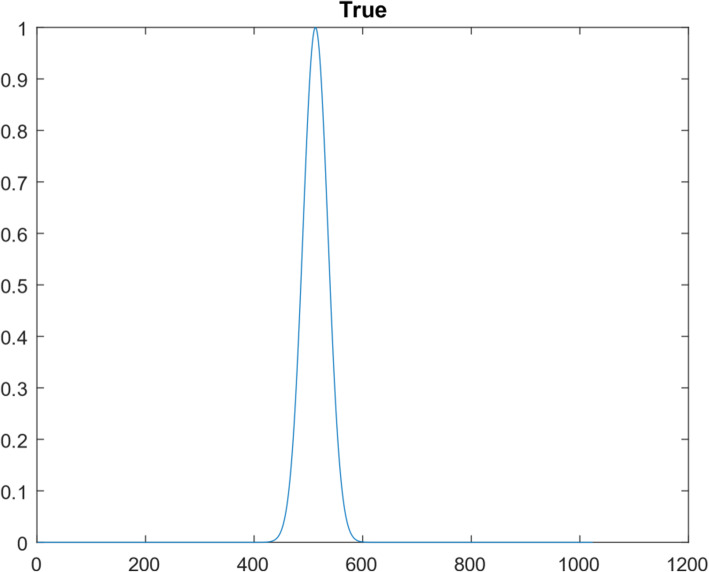
The true Gaussian function

**Fig. 11:**
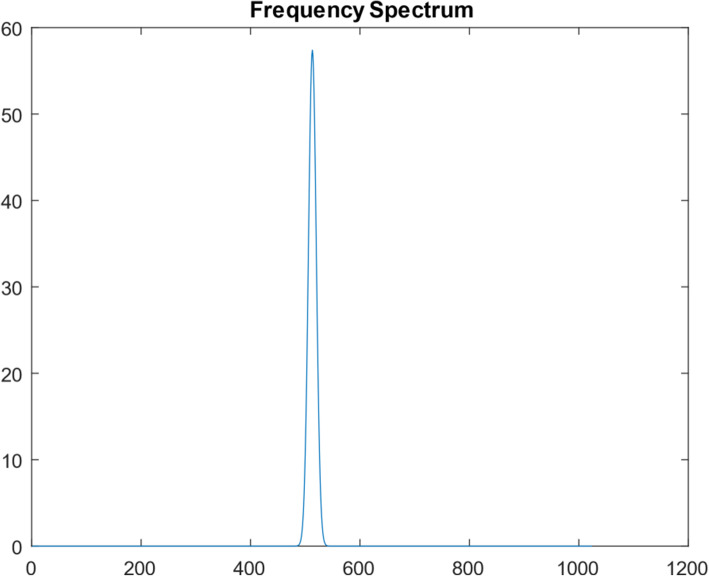
The frequency spectrum of the true Gaussian function

**Fig. 12:**
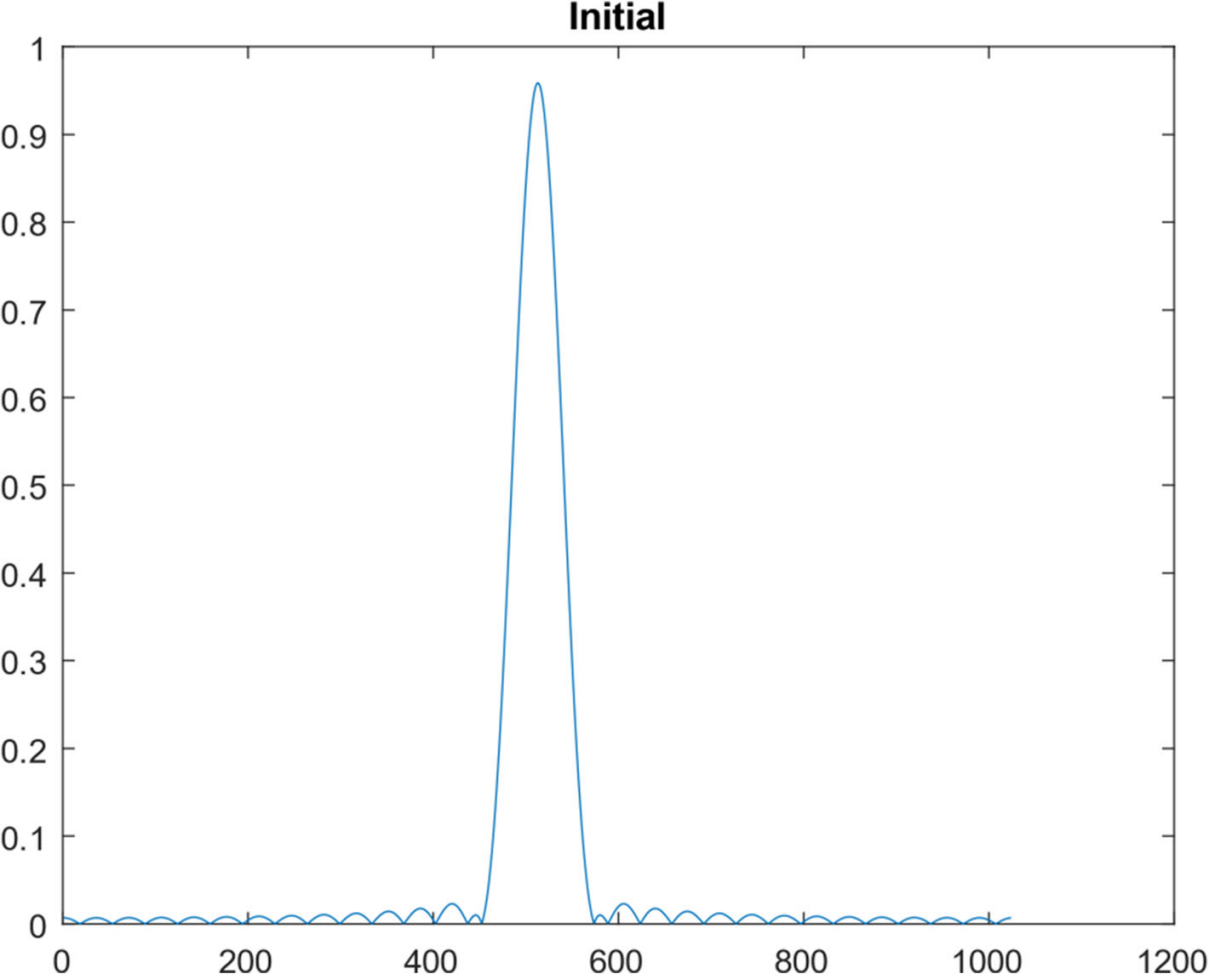
The spatial-domain function obtained from the lowest 30 frequency components of the Gaussian function

**Fig. 13:**
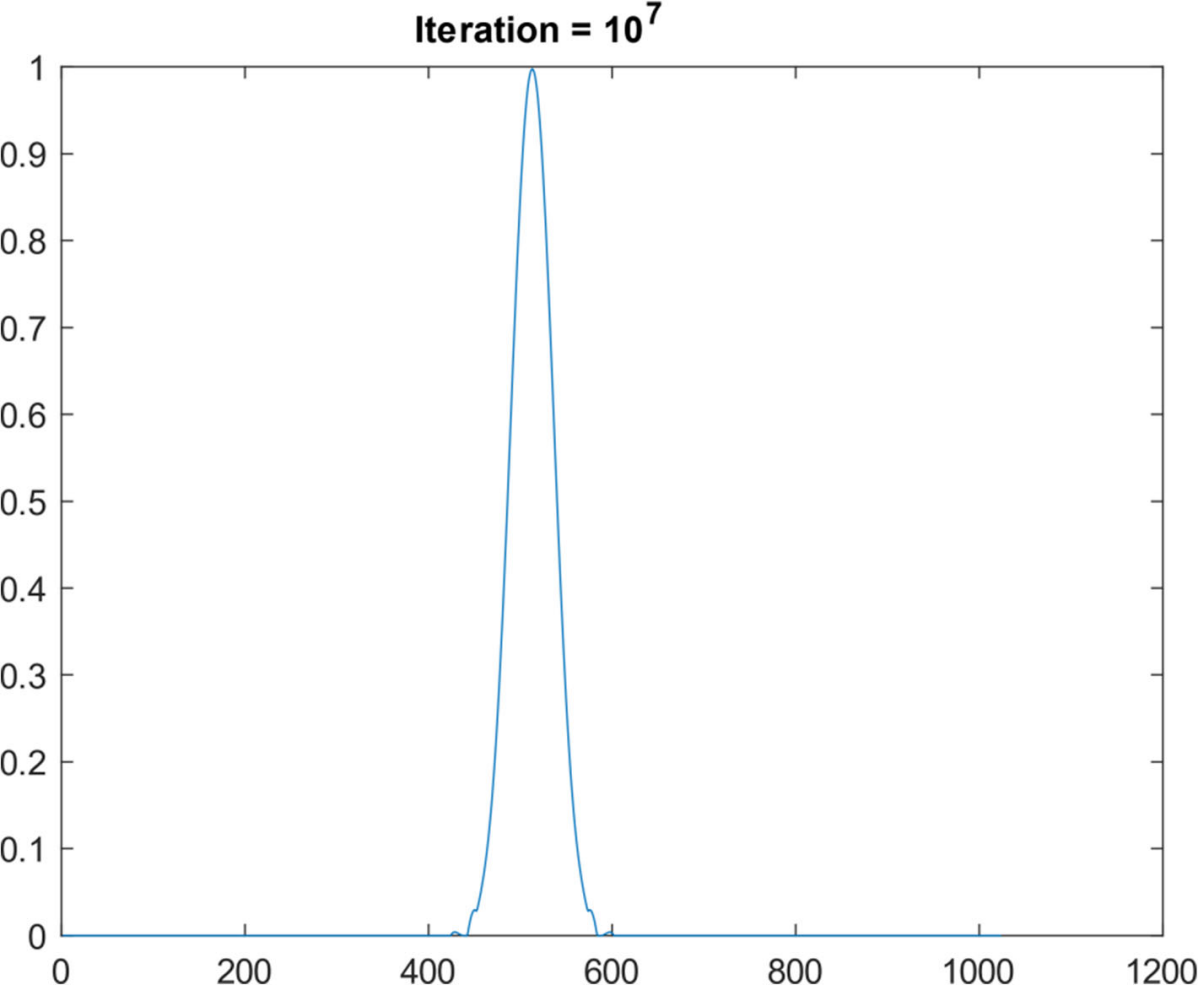
Restored the Gaussian function after 10^7^ iteration of suggested algorithm

**Fig. 14:**
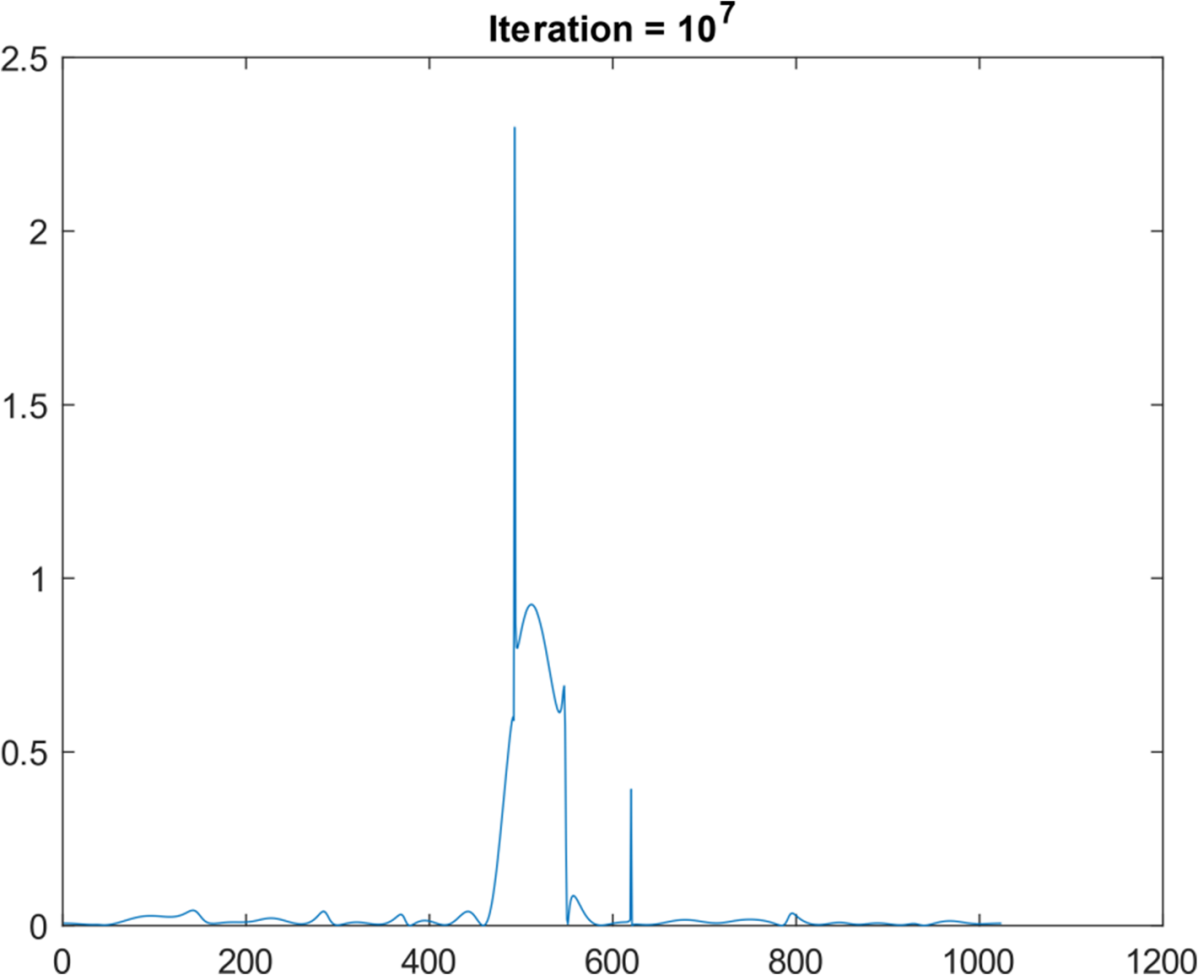
Restored the Gaussian function after 10^7^ iteration of suggested algorithm using noisy data

**Table 4:** MSEs for the 1D Gaussian curve study with noiseless data

Iteration number	MSE
Initial	1.4 × 10^− 4^
10^2^	1.7 × 10^−5^
10^4^	6.3 × 10^−6^
10^6^	2.2 × 10^− 6^
10^8^	1.4 × 10^−6^

The restoration of the first curve used 30 measurements in the frequency domain; the restoration of the first curve used 15 measurements in the frequency domain. It is interesting to notice that the restoration of the second curve is much more accurate than the first curve, by using half amount of measurements.

When comparing the frequency spectra Fig. 6 vs Fig. 11, the spectrum in Fig. 11 is smoother, while the spectrum in Fig. 6 has many sharp oscillations. This observation indicates that the ill-condition of the function extension depends on the function itself.

All computer simulations were performed on a Linux server with an Intel® Xeon® CPU, 2.40 GHz, and a RAM of 128 GB. The algorithm has a slow convergence rate and does not converge to the true values. The algorithm is terminated when a specified iteration number is reached. The computation times are reported in Tables 1 and 2.

## Discussion

To our knowledge, the previous algorithms assumed unmeasured data to be zero. In other words, the results of the previous algorithm are our initial images in the proposed algorithm. Ref. [10] made an attempt to estimate the unmeasured data through over-sampling in the measured region. However, the method in ref. [10] does not apply here, because our data is not over-sampled in the measured range.

After a large number of iterations, the results are not much different from the initial data. This implies a very slow convergence rate if the algorithm converges at all. This can be considered as an ill-condition effect. It is an open question if there exists a strategy to improve the ill-condition without measuring more data. Measuring more data is an obvious method to make a problem less ill-posed.

## Conclusions

We propose an iterative algorithm trying to extend the measured frequency components to unmeasured frequency components. The algorithm is in the POCS form, alternating between the spatial and frequency domains. In the frequency domain, it enforces the measured frequency components. In the spatial domain, it enforces the real and nonnegativity constraints.

The computer simulations imply that the convergence rate of this iterative algorithm is very slow. A very slow convergence rate is a sign that the problem of the analytic continuation is severely ill-posed [6]. Due to the slow convergence rate, there are not many changes and improvements over iterations. In the noiseless cases, the final image looks similar to the initial image; almost no high frequency components are recovered. When there is little noise, the results are too noisy to be useful. The ill-condition nature of the problem depends on the function itself. For some better-behaved functions, more accurate results can be obtained with fewer measurements than some worse-behaved functions. Complete recovering the unmeasured data seems hopeless with the methods we know so far. The problem is still open.

## Data Availability

Not applicable.

## Appendix

Verification that the *F*(*z*) defined in Formula (3) is an entire function.

Here, the *F*(*z*) defined in Formula (3) is rewritten as

$$ F(z)=\underset{-A}{\overset{A}{\int }}f(t){e}^{- izt} dt\kern6.16em \hspace{0.28em}\kern0.28em \hspace{0.28em}\kern0.28em \hspace{0.28em}\kern0.28em \hspace{0.28em}\kern0.28em \hspace{0.28em}\kern0.28em \hspace{0.28em}\kern0.28em \hspace{0.28em}\kern0.28em \hspace{0.28em}\kern0.28em \hspace{0.28em}\kern0.28em \hspace{0.28em}\kern0.28em \hspace{0.28em}\kern0.28em \hspace{0.28em}\kern0.28em \hspace{0.28em}(A1) $$

Let *z* = *x* + *iy*, Formula (A1) becomes

$$ F(z)=u\left(x,y\right)+ iv\left(x,y\right)=\underset{-A}{\overset{A}{\int }}f(t){e}^{yt}\cos (xt) dt-i\underset{-A}{\overset{A}{\int }}f(t){e}^{yt}\sin (xt) dt\kern0.84em (A2) $$

where

$$ u\left(x,y\right)=\underset{-A}{\overset{A}{\int }}f(t){e}^{yt}\cos (xt) dt\kern0.28em (A3) $$

$$ v\left(x,y\right)=-\underset{-A}{\overset{A}{\int }}f(t){e}^{yt}\sin (xt) dt\kern0.28em (A4) $$

It is straightforward to verify that

$$ \frac{\partial u\left(x,y\right)}{\partial x}=-\underset{-A}{\overset{A}{\int }} tf(t){e}^{yt}\sin (xt) dt\kern0.28em (A5) $$

$$ \frac{\partial v\left(x,y\right)}{\partial x}=-\underset{-A}{\overset{A}{\int }} tf(t){e}^{yt}\sin (xt) dt\kern0.28em (A6) $$

$$ \frac{\partial u\left(x,y\right)}{\partial y}=\underset{-A}{\overset{A}{\int }} tf(t){e}^{yt}\cos (xt) dt\kern0.28em (A7) $$

$$ \frac{\partial v\left(x,y\right)}{\partial x}=-\underset{-A}{\overset{A}{\int }} tf(t){e}^{yt}\cos (xt) dt\kern0.28em (A8) $$

Formulas A5-A8 satisfy the Cauchy-Riemann equations

$$ \frac{\partial u}{\partial x}=\frac{\partial v}{\partial y}\kern2.80em (A9) $$

$$ \frac{\partial u}{\partial y}=-\frac{\partial v}{\partial x}\kern2.80em (A10) $$

## Abbreviations

1D: One dimensional
2D: Two dimensional
MSE: Mean squared error
POCS: Projections onto convex set
